# Pediatric Participant Retention Rates in a Longitudinal Malaria Immunology Study

**DOI:** 10.4269/ajtmh.21-1052

**Published:** 2022-04-18

**Authors:** Anushay Mistry, Boaz Odwar, Fredrick Olewe, Jonathan Kurtis, Ann M. Moormann, John Michael Ong’echa

**Affiliations:** ^1^ University of Massachusetts Chan Medical School, Worcester, Massachusetts;; ^2^ Kenya Medical Research Institute, Kisumu, Kenya;; ^3^ Brown University, Providence, Rhode Island

## Abstract

The resurgence of drug-resistant *Plasmodium falciparum* parasites continues to motivate the development of a safe and efficacious malaria vaccine. Immuno-epidemiologic studies of naturally acquired immunity (NAI) have been a useful strategy to identify new malaria vaccine targets. However, retention of pediatric participants throughout longitudinal studies is essential for gathering comprehensive exposure and outcome data. Within the context of a 3-year cohort (*N* = 400) study involving monthly finger prick and bi-annual venous blood sample collections, we conducted qualitative surveys to assess factors impacting participant retention. Phase 1 was conducted 3 months after enrollment in July 2018 and phase 2, 12 months later. In phase 1, 236 parents/guardians participated in focus groups and three withdrawn participants and 10 community health volunteers (CHVs) in key informant interviews. Qualitative analysis indicated overall satisfaction with the study, with 61.8% (136/220 respondents) reporting no concerns. Focus group discussants associated attendance with benefits such as improved access to comprehensive healthcare services. Community health volunteers reported concerns over village rumors of inappropriate use of blood samples and dangers associated with venous blood draws. Phase 2 involved 205 parents/guardians and revealed continued satisfaction, with 46.3% (95/205) identifying no concerns, but expressed increasing worries regarding the amount of venous blood sample. This concern was reflected in an uptick of missed visits when venous blood samples were scheduled. Future studies will address parental concerns to determine whether community engagement and education measures increase study retention until completion.

## INTRODUCTION

Despite numerous advancements in control and treatment over the past decade, malaria remains a significant health burden throughout Africa. Fifteen countries in Africa represent 80% of the global burden of malaria, with Kenya bearing 8% of estimated cases in Eastern Africa.^1^ People living in malaria-endemic areas such as western Kenya develop immunity over time. This naturally acquired immunity (NAI) develops in response to repeated exposure to infectious mosquito bites and ultimately results in the development of antimalarial antibodies. These antimalarial antibodies protect against high parasitic burden associated with severe clinical malaria but do not necessarily protect against repeated infections.^2^ Thus, immunologically-naive children face the brunt of malaria morbidity and mortality. In a surveillance study of clinical and symptomatic parasitological positivity rates of all malaria-suspected patients and school children from 2015 to 2016, Kapesa et al found blood stage positivity ranged from 6.4% at epidemic prone sites to 38.3% at the hyperendemic site.^3^ Children aged 5–14 years showed the highest (45%) parasitemia rate with over 60% positive in holoendemic settings. Malaria remains the major cause of hospital consultations in western Kenya, accounting for 47% of hospital admissions.^3^ This is similar to the 33.5% prevalence of malaria in the Democratic Republic of Congo, with an under five mortality rate of 147 deaths per 1,000 live births.^4^

Even though antimalarial drugs are widely used to cure infections, the resurgence of drug-resistant *Plasmodium falciparum* malaria parasites continues to motivate work toward developing a safe and efficacious malaria vaccine. The leading malaria vaccine candidate RTS,S has a wide range of efficacy against clinical malaria in young children, as evidenced by the phase III clinical trial, which showed a protective efficacy rate ranging from 22.0% to 74.6% depending on geographical location.^5^^,^^6^ This efficacy additionally has been shown to decrease over time, further highlighting the need for alternative parasite targets and vaccination strategies.^7^ Immuno-epidemiologic studies of NAI have proved to be a useful strategy in identifying new malaria vaccine antigens. Previous experiments classified parasite targets of NAI antibodies as well as the *P. falciparum* antigens associated with acquired resistance.^8^ Antibodies to parasite antigens such as *P. falciparum* schizont egress antigen-1 (PfSEA-1) and *P. falciparum* glutamic-acid-rich protein (PfGARP) have shown efficacy in decreasing parasite replication and parasitemia.^9^^,^^10^ Additional candidate antigens thus must be tested for potential inclusion in a multi-antigen malaria vaccine and may lead to improved efficacy through synergy of parasite antigens such as PfGARP and PfSEA-1.

To identify and validate novel vaccine candidates based on NAI requires large, longitudinal cohorts. One challenge faced by studies involving pediatric cohorts is avoiding high attrition rates. Retention of participants throughout longitudinal studies is essential for gathering comprehensive repeated malaria exposures and clinical outcomes data.^11^ A systematic review of participant retention studies identified strategies such as collecting updated and alternative contact information, after-hours phone calls, culturally sensitive staff, flexible study event scheduling, clinic visit transportation newsletters, drop-in home visits, and cell phone reimbursements.^12^^,^^13^ Universal to studies with high retention rates was the use of culturally informed retention strategies and incentives.^14^^,^^15^ Also noted by multiple studies was the importance of continuous reevaluation of cohort retention strategies throughout the duration of the study.^13^ Although the use of multiple strategies has been demonstrated as effective for adult participants, retention of pediatric participants is especially challenging.^16^ In two studies conducted in South Africa, the use of lay community health workers was shown to be particularly effective in participant retention.^17^^,^^18^ In their study of retention of children on antiretroviral treatment, Grimwood et al. noted that community health workers provided adherence and psychological support for children’s caregivers and undertook home visits to ascertain household challenges potentially impacting child adherence.^19^ These studies exemplified the necessity for community-based support strategies to improve retention in studies of child participants.

Based on a partial understanding of the challenges encountered for prospective pediatric cohort studies, the current study used multiple methods to examine factors that impact participant retention rates. The parent project evaluating NAI to malaria enrolled 400 pediatric study participants and asked their parents to allow them to participate for up to 3 years. The goal of the immunology study is to analyze the maturation of follicular helper T cells and the development of antibodies specific to novel malaria vaccine candidates. We conducted qualitative surveys, focus groups, and key informant interviews to identify and assess factors that positively and negatively impacted participant retention rates.

## MATERIALS AND METHODS

**Figure 1.**
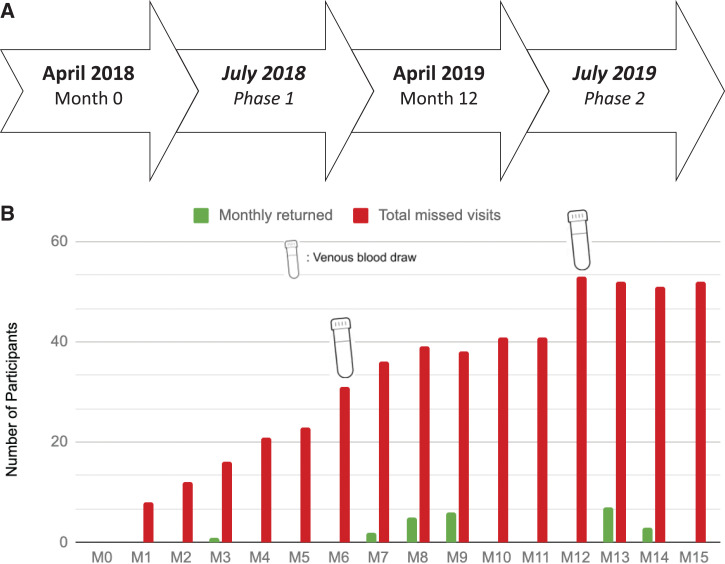
Study timeline and blood collection schedule. (**A**) Three-year immunology cohort study of 400 children started in April 2018. Nested within was a study to determine reasons for study participation and retention, conducted in two phases, in July 2018 and 1 year later. (**B**) Graph illustrating number of participants missing corresponding monthly visit and type of blood collection scheduled as well as number of returned participants who presented for corresponding monthly visit. Finger prick (small volume) of blood was collected each month except month 6 (M6) and M12 when venous blood samples were collected for cellular immunity studies. “*Monthly returned*” (gray bar) depicts the number of participants who missed one or more prior visits but presented for the corresponding monthly visit. “*Total missed visits*” (black bars) depicts the number of participants who missed the corresponding monthly visit added to the number of participants who missed the prior monthly visit minus the “*monthly returned*.” This figure appears in color at www.ajtmh.org.

In April 2018, 400 children (2–7 years of age) were enrolled in a cohort study involving monthly finger pricks and biannual venous blood sample collections of 10 mL (Figure 1A). Participants were scheduled to come to the designated research area at a local clinic on a specific day according to their assigned community health volunteer (CHV), with roughly two CHVs and 75 children presenting per day. Phase 1 data were collected 3 months after initial enrollment, in July 2018, during the week of scheduled monthly visits. Phase 2 data were collected 15 months after enrollment, in July 2019 (Figure 1A). Both phases 1 and 2 data were gathered during monthly visits that did not coincide with venous blood sample collections. In both phases, data were obtained from parents or guardians of participants using focus groups guided by semi-structured questionnaires (Supplemental Material A) and from CHVs using key informant interviews (Supplemental Material B). Participants who missed their monthly visit were contacted by their assigned CHV the next day and questioned using individual interviews during a home visit to determine reasons for the participant’s missed visit. Focus groups and key informant interviews were conducted in accordance with semi-structured group or interview guides, respectively. Individual interviews with the CHVs were conducted in English, whereas focus groups and individual interviews with withdrawn participants were conducted in the local language of Dholuo and translated to English by the overseeing CHV.

In both phases 1 and 2, parents or guardians of the retained enrollees were asked to participate in focus groups to determine the factors that positively or negatively affected retention in the study. Qualitative data were gathered through open-ended response questions and quantitative data through questions asking for show-of-hand responses. Parent questionnaires elicited information on travel for monthly visits, motivations and concerns for study participation, and access to healthcare prior to the study. To increase parent response rates to open-ended questions, data from phase 1 were analyzed to generate multiple-choice options for certain questions in the phase 2 questionnaire. For example, in response to inquiries regarding motivations for study enrollment, given options included, but were not limited to, “optimization of healthcare access” and “supportive of study goals.” Fahui Wang defines healthcare accessibility as the relative ease by which services can be reached from a given location and optimization of healthcare accessibility as improvement in the distribution of healthcare services to maximize service coverage and minimize barriers to access.^20^ After completion of the focus groups, CHVs were interviewed individually to solicit their impressions of factors impacting study retention. The key informant interviews of CHVs included open-ended questions about prior experience with research studies, training received for current study, and retention strategies implemented to discourage participant attrition as well as the CHVs’ opinion of why participants joined and/or withdrew from the study.

After completion of all focus groups and CHV key informant interviews each day, the final numbers were tallied and each participant who failed to present for their monthly visit was identified by the CHVs. The study team would then visit the participants who missed their monthly visit and conduct key informant interviews to attempt to elicit the reason for the participant’s failure to present. Based on this information, participants were categorized as either having withdrawn from the study or having missed the visit with intention to remain enrolled in the study. Participants who reported to their CHV that they did not want to continue participating in the parent immunology study were invited to participate in interviews to elicit the reason for their withdrawal. The interviews were conducted in person during home visits by the study team. Of note, some participants who initially withdrew from the study presented again at a later point for a monthly visit and were allowed to resume study participation to maintain good will with the community.

After phase 2 of this study, participant homesteads were mapped using Arc Geographical Information System (ArcGIS), a geographical information system made by the Environmental Systems Research Institute (ESRI). The mapping was conducted by the CHVs and other members of the study team during scheduled home visits to participants’ villages. This mapping data enabled us to estimate the potential distance each participant traveled to reach the study clinic for research visits. The mapping data along with all other quantitative data collected in this study were graphed and analyzed using GraphPad Prism 9. χ^2^ analyses were conducted using Prism 9 to determine the significance of trends between phases 1 and 2 of the study.

Ethical approval was received from the Scientific and Ethics Research Unit (SERU) at the Kenya Medical Research Institute (KEMRI).

## RESULTS

### Overall study population and attrition.

Our study included a total of 268 parents or guardians; of the 400 children enrolled, 132 (33.3%) shared a household with one or more study participants. Of the 382 participants for whom sex is known, 192 subjects are male and 190 subjects are female. In phase 1, during the week of scheduled monthly visits, focus groups of 236 (88.1%) parents or guardians of participants as well as key informant interviews with 10 CHVs and three withdrawn participants were conducted. Phase 2 consisted of focus groups with 205 (87.2%) of 235 parents or guardians of participants still enrolled as well as key informant interviews with nine of the 10 CHVs and four withdrawn participants.

### Focus groups.

**Figure 2.**
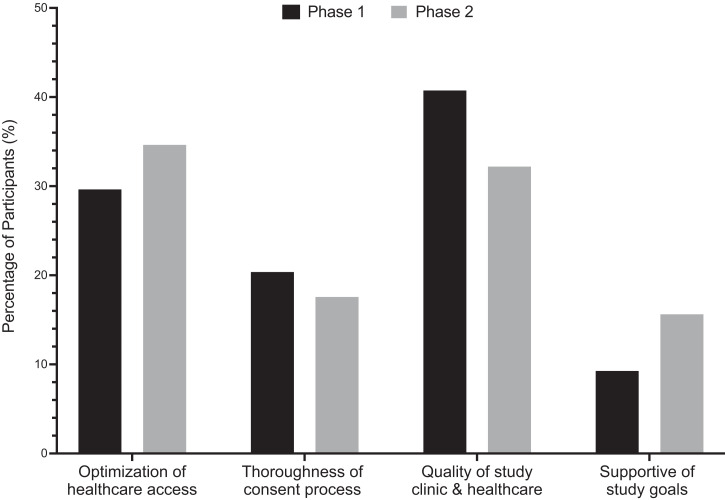
Primary reason for study participation. The percentage of participants identifying given reason as primary motivation for study enrollment in phase 1 (black bars) or continued participation in phase 2 (gray bars) based on focus group responses. Depicts an increase in reported satisfaction with optimized access to healthcare from 29.6% in phase 1 to 34.6% in phase 2 and in reported support of the goals of the parent study from 9.3% in phase 1 to 15.6% in phase 2.

In phase 1 of the study, qualitative analysis of focus group responses revealed that participants associated enrollment in the study with benefits such as improved access to comprehensive health services and noted a deep appreciation for the thorough and extensive consent process. The main reason for enrollment identified by the study participants in phase 1, however, was the high quality of the healthcare provided by the clinical officer assigned to work with the research study (Figure 2). One parent noted “the children’s health, it is better when they are in the study.” In phase 2, however, the major reason for continued enrollment in the study was identified by participants to be the optimization of access to healthcare (Figure 2). Thirty-two percent of participants (66/205) continued to list the higher quality of healthcare but a larger percentage, 34.6% (71/205) indicated the ease of accessing quality healthcare as the main reason for continued enrollment (Figure 2). A χ^2^ test of independence, however, showed no significant relationship between the reasons with study participation in phases 1 and 2, χ^2^ (3, *N* = 205) = 2.630, *P* = 0.4523.

**Figure 3.**
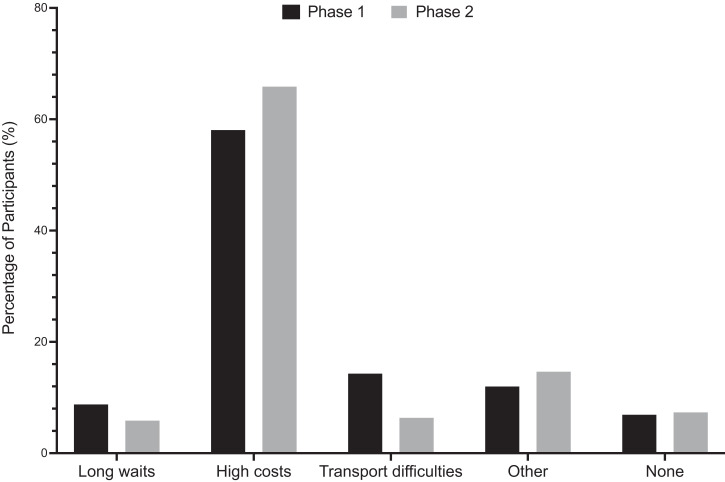
Past challenges accessing healthcare. The percentage of participants in phase 1 (black bars) and phase 2 (gray bars) identifying given reason as primary challenge accessing healthcare prior to study participation. Depicts an increase in reported challenges accessing healthcare because of high costs from 58.1% in phase 1 to 65.9% in phase 2.

Prior to participation in the study, participants report having experienced difficulties accessing transportation to healthcare clinics, long wait times to see a healthcare provider, and, most significantly, high costs of provider visits and medications (Figure 3). As stated by one parent, “before the study, even to see the doctor we need money, and sometimes the doctor, they don’t have the medications; now, we can see the doctor and receive the medications to treat the babies all at the same time, and for free!” Additionally, many more participants (15.6%) stated their support for the goals of the study during phase 2, possibly indicating an increase in understanding of the study goals over time (Figure 2). In reply to inquiries regarding motivation for participation in the study, parents spoke primarily of the burden of malaria on their community and their desire to further any research that could help alleviate this burden. A χ^2^ test of independence showed a borderline significant relationship between the reported challenges to healthcare access in phases 1 and 2, χ^2^ (4, *N* = 205) = 9.207, *P* = 0.0561.

**Figure 4.**
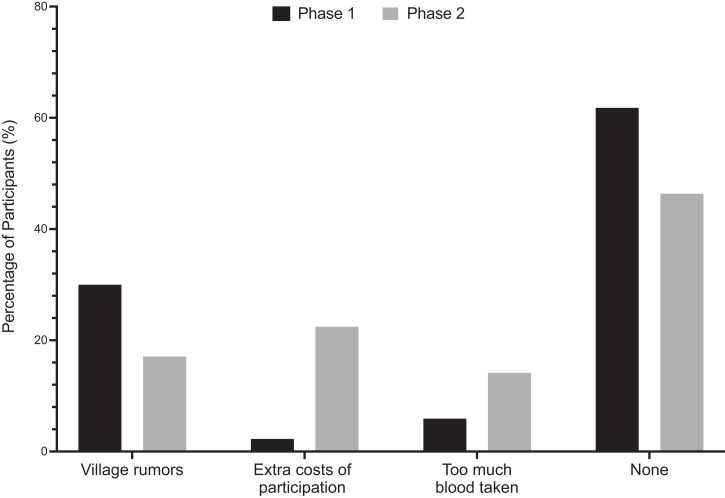
Primary concern after study enrollment. The percentage of participants identifying given reason as primary concern for study enrollment in phase 1 (black bars) or continued participation in phase 2 (gray bars). Depicts increase in reported concerns regarding extra costs of study participation from 2.27% to 22.4% and decrease in reported concern regarding village rumors from 30% in phase 1 to 17.1% in phase 2.

Concerns for continued enrollment in the study were consistent between participants and CHVs in phase 1 of the study. Four CHVs and 30% (66/220) of focus group participants reported that community members who were not involved in the study perpetuated rumors of inappropriate use of blood samples and dangers associated with venous blood draws (Figure 4). One parent noted a common rumor stating, “people in the community say they bring their children here to give their blood to ‘devil worshippers’ who sell the blood.” Another concern expressed was the volume of blood drawn, noted by 5.9% (13/220) of focus group participants and one CHV. Complaints of insufficient incentives provided in return for participation in the study were stated by two CHVs but not expressed by any focus group participants. However, overall, the majority of focus group participants, 61.8% (136/220) reported no concerns with the study (Figure 4).

In phase 2, the complaints shifted away from the rumors of the village members, with only 15% (35/205) continuing to report village rumors as their primary concern (Figure 4). The majority of parents and guardians, 22.4% (46/205), reported their primary concern to be the extra costs of participation in the study, such as the rising costs of transportation or the lost income from a missed day of work. As stated by one parent, “the people who are bringing them here, the motos, add price because they know we will be reimbursed.” Additionally, more parents 14.1% (29/205) mentioned the amount of blood being drawn during venous collections as a concern of continued enrollment in the study (Figure 4). One parent reported, “the children, they are more tired after giving blood and do not play as much as on other days.” This was an increase from phase 1, when only 5.9% (13/220) had reported similar concerns. Although half as many parents noted concerns over village rumors and twice as many reported concerns over the amount of blood taken, the majority, 46.3% (95/205), reported no concerns (Figure 4). A χ^2^ test of independence showed a significant relationship between the concerns with study participation in phases 1 and 2, χ^2^ (3, *N* = 205) = 55.39, *P* < 0.0001.

### Key informant interviews.

Key informant interviews with CHVs of the study gave insights into strategies for participant retention as well as the CHV’s impressions of participant motivations for continued participation in the study. During phase 1, common themes of retention strategies included an emphasis on communication, involvement of the participant’s communities as well as material incentives, and monetary reimbursements (Table 1). The major reasons for participant enrollment noted by the CHVs were to become more aware of and able to advocate for their children’s health and to learn more about the problems caused by malaria in their communities. One CHV noted, “In the study people are getting more skills and knowledge to help to keep their children safer.” Overall, the participant motivations suggested by CHVs strongly matched those reported by participant focus groups. Both CHVs and participants identified the increased ease of accessing healthcare and the higher quality of care as major reasons for participation in the study. The main difference between CHVs’ perception of participant motivations and participant’s actual motivations was the importance of the goals of the study. Six of 10 CHVs listed support for the goals of the study as a reason for participant enrollment, whereas only 10% of participants actually identified belief in the goals of the study as motivation.

**Table 1:** Implementation strategy themes for participant retention

Theme	Example of implemented strategy
Community involvement	Involvement of participants/parents in execution of study protocol
	Recruitment of CHVs from study participants’ communities
Emphasis on communication	Direct communication between principal investigators and study participants’ community prior to participant enrollment
	Regular reminders of monthly visits
	Weekly CHV visits with wellness checks for study participants
Incentives for remaining in study	Transport reimbursements at each monthly visit
	Material incentives of soap and milk at each monthly visit

CHV = community health volunteer.

Phase 2 revealed similar results, with CHVs pointing primarily toward perceived benefits of participating in research studies as the major reason for participant enrollment. However, as opposed to phase 1, only one out of the nine CHVs interviewed listed belief in the study goals as a reason for participation. Three CHVs noted the specific benefits of better health in the children because of easier access to healthcare and the perceived superiority of the healthcare provided when part of a research study. As stated by one CHV, “according to my community, there are some who are interested in KEMRI and there are some that are not because of the noise.” When asked to clarify, this CHV identified community rumors stating KEMRI to be selling the participants’ blood as the “noise” and noted that some parents who believe in these rumors refuse to participate in any studies associated with KEMRI.

### Withdrawn participants.

In phase 1, withdrawn participants were individually interviewed to determine the major causes for attrition or missing their monthly visit. Interviews were held at the participant’s homes during follow-up visits and conducted by the respective CHV. Participants who missed the study visit but remained enrolled in the study were interviewed to determine the reason for their missed visit. A distinction was made between participants who withdrew fully from the study, those who intentionally chose to skip a particular monthly visit and those who randomly missed a monthly visit. Through these key informant interviews in association with review of the study protocol, there appears to be a trend between increased attrition for the monthly visits during which venous blood was drawn (months 6 and 12) and increased numbers of participants returning for the subsequent visits and thus remaining enrolled in the study (Figure 1B).

Of the three withdrawn participants during the month of July, two were siblings from the same household and the third resided within the same sublocation. Another commonality between the two families was that both children, when sick, were brought to the local dispensary rather than the study clinic. When asked for the reason for their preference for the local dispensary, one mother cited the distance to the study clinic as a barrier for access. However, the other mother indicated a preference for the local dispensary because of a loss of trust in the study clinic. The mother reported that at the first monthly visit, her child was sick and given medication but did not get better. Henceforth, the mother believed she was receiving subpar medical care and chose instead to bring her child to the local dispensary. With regard to the cause for study withdrawal, interviews with both mothers identified the cause for attrition to be a concern over the amount of blood taken during the monthly visits. In the first case, the child reported dizziness following the initial blood draw and subsequent reports of anemia by the local dispensary led the mother to withdraw the participant and sibling. At the second household, despite no adverse symptoms after sample withdrawal, the mother withdrew her child following reports of community member concerns.

Phase 2 both confirmed previously identified concerns while revealing new causes of attrition. Of the 11 participants who withdrew from the study in the year after phase 1, seven participants withdrew because of relocation away from the study site. As confirmed with their respective CHVs, these seven participants reported no concerns with the study and only withdrew following relocation greater than 2 hours away from the study clinic. This contrasts with phase 1 data, which identified the amount of sample as the major reason for attrition. In phase 2, four participants cited concerns over the amount of sample taken during venous blood draws as their reason for attrition. Interestingly, these participants were all from the same village and the CHV assigned to this village had previously reported village rumors regarding the amount of blood being taken.

To assess distance from home to clinic as a possible reason for study attrition or number of missed clinic visits, the average distance from participant homes to study clinic was calculated. It was determined that the average distance traveled for retained participants was 5.67 km (0.11–11.7 km) as opposed to the distance traveled by withdrawn participants, which was 3.74 km (0.25–7.95 km). These differences were not significantly different and are consistent with the findings of focus group discussions, which did not identify distance as a barrier to study participation.

## DISCUSSION

Immuno-epidemiologic studies of NAI are essential to the development of a safe and efficacious malaria vaccine but introduce the challenge of minimizing participant attrition, especially when involving pediatric cohorts.^19^ Maximal participant retention rates are essential throughout longitudinal studies to gather comprehensive outcomes data. Within the context of a 3-year, 400 children cohort study, we conducted baseline and follow-up qualitative surveys, focus groups, and key informant interviews to assess factors impacting participant retention rates within the first year of the study. Overall study retention rate in phase 2 was 349 out of the 400 (87.2%) participants initially enrolled. Our baseline surveys and interviews from both phases 1 and 2 indicated overall satisfaction with study participation, with a 15.5% decrease from phase 1 to phase 2 in the number of parents reporting no concerns with the study. Analysis of the data mapping participant homesteads and the distance to the study clinic revealed no significant correlation of distance traveled to study clinic and participant attrition. χ^2^ analyses revealed a significant relationship (*P* < 0.0001) between phases 1 and 2 data of concerns with study participation. The main concern reported by parents shifted from village rumors (30%) in phase 1 to extra costs of participation (22.4%) in phase 2. Additionally, the number of parents reporting concerns over the amount of blood taken doubled from phase 1 (5.9%) to phase 2 (14.1%). Interestingly, this shift from phase 1 to phase 2 correlated with an increase from 9.2% to 15.6% in the number of participants identifying support of study goals as their main reason for continued study participation. These trends suggest a perceived deepening in both the participants’ and the communities’ understanding of study goals but highlight the need for continued discussions regarding the importance of the venous blood collection for cellular immunity assays.

The importance of low attrition rates for longitudinal pediatric cohorts has led to numerous studies evaluating various retention strategies and assessing their efficacy. The most commonly used retention strategies and implementation approaches with high retention rates seem to be individual reminders, an emphasis on incentives and individualized scheduling strategies.^11^^–^^13^ One commonality of studies with high retention rates was their emphasis on culturally informed and sensitive retention strategies, tailored to the specific study population.^15^^,^^18^ This supports the use of CHVs in our study as each CHV was identified from their home village and assigned to participants from the same or nearby villages. Thus, each CHV was well versed in the culture and customs of the participants in their assigned study site and able to tailor their retention strategies appropriately. Gappoo et al. further identified commonly used retention strategies specifically in South Africa and Zimbabwe while additionally noting common reasons for attrition.^17^ Consistent with our results, the most common reasons for participant withdrawal were participant relocation, community rumors, and familial disapproval.^17^ Grimwood et al. further investigated the value of culturally informed retention strategies by comparing outcomes between South African children on antiretroviral therapy with and without community-based adherence support from patient advocates.^18^ The results from this study further confirmed our results, showing that community-based adherence support effectively improved individual participant retention.^18^

These results should be interpreted with caution and highlight a few limitations that could be addressed by future research. One major limitation inherent to many international research efforts is a lack of fluency in the participants’ native language, Dholuo. Focus groups and individual interviews with withdrawn participants were conducted in Dholuo by CHVs and subsequently translated into English for data gathering, thus introducing the possibility of biased translation. This could be addressed in future studies through the use of a second, independent person fluent in both English and Dholuo to verify translations. Additionally, the focus group questionnaires and key informant interviews conducted in this study relied upon participant reporting, thus leading to the potential for acquiescence bias. This study was also subject to limitations because the inherent nature of qualitative research as question-based and the difficulty of investigating causality through qualitative data. Finally, although the kilometers traveled from participant homes to the study clinic were measured, these raw distances do not necessarily correspond to ease of travel because of local topography and subsequent transportation difficulties. Public transportation in Kenya consists primarily of *tuktuks*, *mototaxis*, and *motobikes*, each of which have differing capabilities to traverse terrain. Localities with difficult topography, therefore, may be closer in kilometers but have fewer transportation options, making it a longer trip. Thus, a complete understanding of ease of access to the study clinic would necessitate more thorough investigation of travel time and transportation methods correlated with travel distance.

Future studies are warranted to monitor satisfaction and determine the source of ongoing and any new community-based concerns. Further research could help to identify the cause of such rumors as well as methods through which they can be prevented or dispelled. The persistence of these rumors over the course of the study may also warrant additional community education to alleviate study-related fears. Concerns regarding the amount of blood volume, for example, could be addressed through education targeted at CHVs and parents on the minimal risks of blood draws of this amount. Additional training for CHVs as well as intermittent meetings with the communities-at-large may enable study staff to address the concerns through question-and-answer sessions with influential village members. Such meetings could foster strong community partnerships and continuous dialogues with all village members, not only those enrolled in the study.

## Conclusions

In conclusion, the importance of maximal participant retention rates in immuno-epidemiologic studies with longitudinal designs cannot be overstated. Studies that provide information on methods through which to increase participant satisfaction and continued enrollment over multiple years, especially among pediatric samples, offer a significant contribution to research on NAI as a means to identify new malaria vaccine targets.
